# Mechanical Recycling of Carbon Fiber-Reinforced Polymer in a Circular Economy

**DOI:** 10.3390/polym16101363

**Published:** 2024-05-10

**Authors:** Salem M. Aldosari, Bandar M. AlOtaibi, Khalid S. Alblalaihid, Saad A. Aldoihi, Khaled A. AlOgab, Sami S. Alsaleh, Dham O. Alshamary, Thaar H. Alanazi, Sami D. Aldrees, Basheer A. Alshammari

**Affiliations:** 1 Innovation Parks, King Abdulaziz City for Science and Technology, Riyadh 11442, Saudi Arabia; saldosari@kacst.edu.sa; 2 Enhanced Composite and Structures Centre, Cranfield University, Cranfield MK43 0AL, UK; 3 Advanced Material Technology Institute, King Abdulaziz City for Science and Technology, Riyadh 11442, Saudi Arabia; bmalotaibi@kacst.edu.sa (B.M.A.); kalogab@kacst.gov.sa (K.A.A.); dalshammari@kacst.edu.sa (D.O.A.); thaar@kacst.edu.sa (T.H.A.); sdrees@kacst.edu.sa (S.D.A.); 4 Institute of Space and Earth Science, King Abdulaziz City for Science and Technology, Riyadh 11442, Saudi Arabia; alblehed@kacst.edu.sa (K.S.A.); saldoihi@kacst.edu.sa (S.A.A.); 5 Future Economy Technology Institute, King Abdulaziz City for Science and Technology, Riyadh 11442, Saudi Arabia; salsaleh@kacst.edu.sa

**Keywords:** carbon fiber-reinforced composite, recycling of composite materials, carbon fiber materials, recycling of carbon fiber, finite element analysis (FEA)

## Abstract

This review thoroughly investigates the mechanical recycling of carbon fiber-reinforced polymer composites (CFRPCs), a critical area for sustainable material management. With CFRPC widely used in high-performance areas like aerospace, transportation, and energy, developing effective recycling methods is essential for tackling environmental and economic issues. Mechanical recycling stands out for its low energy consumption and minimal environmental impact. This paper reviews current mechanical recycling techniques, highlighting their benefits in terms of energy efficiency and material recovery, but also points out their challenges, such as the degradation of mechanical properties due to fiber damage and difficulties in achieving strong interfacial adhesion in recycled composites. A novel part of this review is the use of finite element analysis (FEA) to predict the behavior of recycled CFRPCs, showing the potential of recycled fibers to preserve structural integrity and performance. This review also emphasizes the need for more research to develop standardized mechanical recycling protocols for CFRPCs that enhance material properties, optimize recycling processes, and assess environmental impacts thoroughly. By combining experimental and numerical studies, this review identifies knowledge gaps and suggests future research directions. It aims to advance the development of sustainable, efficient, and economically viable CFRPC recycling methods. The insights from this review could significantly benefit the circular economy by reducing waste and enabling the reuse of valuable carbon fibers in new composite materials.

## 1. Introduction

Polymers are among the most consumed and well-known materials of this century. Nevertheless, pure polymers are inadequate for several industrial applications that require extraordinary strength and thermal resistance. Thus, engineers have reinforced polymers with other strong materials to achieve the required performance and properties to meet the requests of several industrial sectors. Carbon fiber (CF) as a unique material has drawn the consideration of scientists due to its outstanding features and characteristics, i.e., light weight, superior mechanical strength at high temperatures with excellent chemical and thermal resistance, great strength-to-weight ratio, low toxicity, non-corrosivity, and recyclable materials [1,2].

CF materials have remarkable electrical, mechanical, and thermal properties. These benefits allow these materials to enhance the properties of CF-reinforced polymer composite (CFRPC) products. CF materials have been developed using several precursors including rayon, oil pitch, and synthetic polymers (i.e., polyacrylonitrile and polyethylene). Polyacrylonitrile is presently considered the first choice for the manufacture of CF materials that are used in important strategic industries such as the aerospace, automobile, and medical industries due to its light weight and great mechanical and chemical properties. This precursor is usually spun up, followed by thermal treatment, including oxidation and carbonization at elevated temperatures. To increase the carbon content in the final CF product, elevated temperatures have been applied to carbonized CF materials [3,4]. However, optimizing the structure and properties of the final CF product does not just occur during the carbonization practice only but during the whole development process, comprising precursor polymerization, spinning, and heat treatment stages [5].

Additionally, CF materials can be classified according to their orientation within the polymer matrices or the length of the fibers (e.g., continuous or discontinuous fiber). Consequently, the application of CFRPCs containing discontinuous CF materials with a high volume rate requires prerequisite anisotropic properties. Conversely, CFRPCs with continuous CF materials are utilized in lower-volume-rate applications, and developed mechanical properties are essential in both directions. It has also been reported that the orientation of fibers affects the final properties and performance of CFRPC products [6,7,8,9].

The cost of CF is considered as the main factor that limits its widespread use in the marketplace and a major contributor to the overall production cost of CFRPC products. For example, about 50% of the production cost of CF materials is associated with producing polyacrylonitrile precursors. For this reason, scientists use cheaper precursor materials such as polyolefin, which offer a huge cost-saving potential for high-volume applications [2,10,11]. Another potential solution to reduce the manufacturing costs of CF is to reuse CF, as it has been reported that recycled CF materials achieve acceptable performance and properties for some industrial requirements [4,12,13,14,15,16].

Hegde et al. [17] emphasized that the cost of CF materials dropped in the 1990s, which led to their utilization in sports goods. They also reported that between 1998 and 2006, the demand for CFRPCs doubled globally, and this demand is expected to grow yearly. It has been reported that the annual worldwide demand for CF has risen to up to 55,000 tons per year [18]. Also, the market for CF materials is predicted to rise from USD 7 to 8.9 billion between 2020 and 2031 with a rate of >8%. The approaching demand from the energy, aerospace, and defense sectors is described by Peijs et al. [10] and Gogoi et al. [12]. Furthermore, Khurshid 2020 et al. [19] estimated the worldwide demand for CF materials and the predicted waste generated from composite materials and end-of-life components in industry.

**Figure 1:**
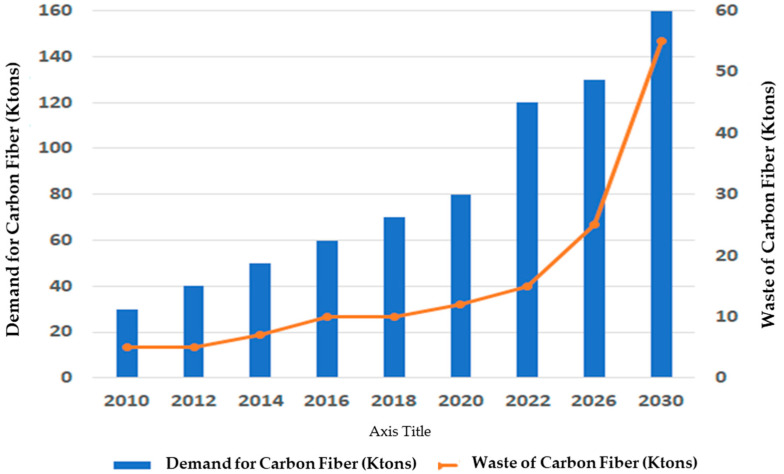
Worldwide demand for CF and estimation of its wastes, adapted from [19].

The global demand for CF and the evaluation of its waste arising from the composite materials industry are given in Figure 1. With the worldwide usage of CF products, concerns that appear are the life cycle of these products, waste disposal, environmental pollution, energy used for manufacturing, and raw material costs. With any recycling goal, the main challenge is reducing manufacturing costs by producing a new product from undesirable waste materials. Waste materials undergo reprocessing to create value-added products. The manufacturing cost of CF has been stated to be EUR ~24–48 per kg for a virgin product and EUR ~13–19 per kg for a recycled one (~40% reduction in the cost). This indicates the relevance of using recycled CF as a reinforcement agent instead of virgin CF in producing composite material products [20]. Consequently, the recycling of CF materials will play a key role in the future to meet these demands. Therefore, the advantages of recycling include obtaining CF materials suitable for remanufacturing and end-of-life sustainability concerns. Hence, recycling unwanted waste materials is encouraged everywhere in the world. In most industries, it is recognized as the solution to many industrial and environmental challenges. Besides the cost-effective benefits of this reprocessing, it also delivers environmental encouragement to consider the entire life cycle of the manufacturing process.

**Figure 2:**
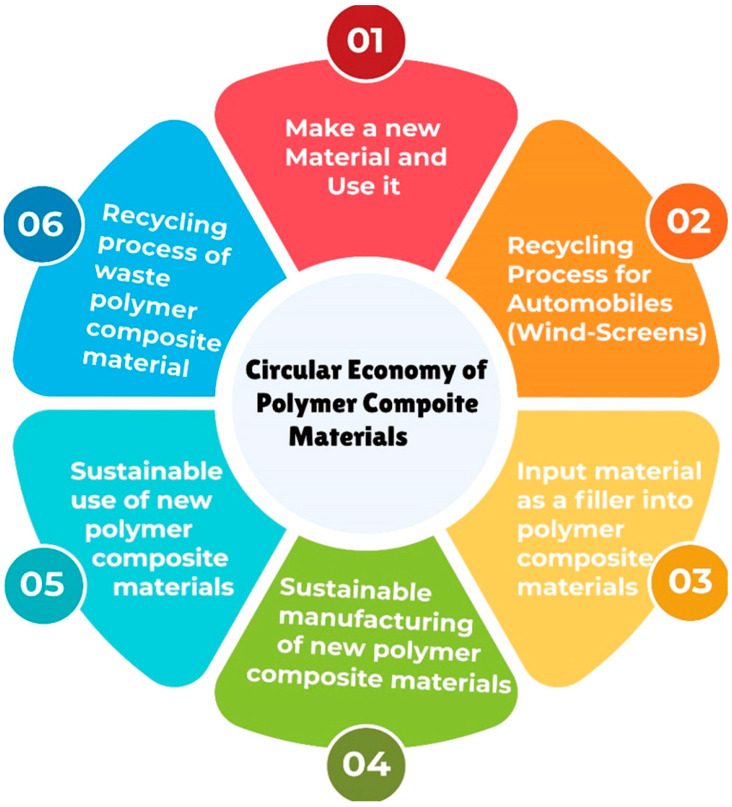
Circular economy model for polymeric composite materials, adapted from [25].

Implementation of zero-waste manufacturing methods (well known as the circular economy) to reduce wastes and reuse resources requires recycling such products after their end-of-life stages [21,22]. A circular economy that has been proposed for polymer composite waste is shown in Figure 2. While these products present important industrial opportunities, their integration into the circular economy poses challenges. This is attributed to their properties and specific characteristics, along with the limited availability of sustainable end-of-life waste management technologies. In spite of these constraints, many technologies associated with circular economy principles (reusing, reducing, recycling) can be applied to develop end-of-life strategies for CFRPCs, offering a concise roadmap towards circularity in industrial applications [23]. A recent review by Zhang et al. [13] discussed in detail the challenges facing the recycling processes of CFRPC waste. They stated that the cost of a new CF product is comparatively high; thus, the recovery of CFs has sound economic advantages.

A study was conducted by Shehab et al. [24], who focus on cost modeling for recycling CFRPCs, encompassing both current state-of-the-art practices and future research. Overall, the recycling of CFRPCs is not executed for economic reasons only, but also to manage the high volume of waste from such materials that is anticipated in the near future. The developed system can be employed to aid early-stage designs and decision-making processes, offering a convenient and rapid means to comprehend and predict recycling costs. The authors reported that the global market size for CFRPCs was estimated to be approximately USD 5 billion in 2019, and it was expected to grow by 10.6% annually, reaching around USD 8 billion in 2024.

While the primary motivation for mechanical recycling is economic, there have been limited efforts to quantify its specific environmental benefits or determine whether it is a preferable option compared to other waste treatments. Evaluating the environmental suitability of a recycling process goes beyond just considering the energy consumption rate; it involves quantifying all potential environmental impacts throughout the recycling chain, encompassing material collection, transportation, byproduct treatment, and the acquisition of usable materials. The success of recycling, and its overall suitability, depend significantly on the type and quantity of virgin CFRPC material available for replacement after the recycling process, as well as the impact of the recovery process itself. Identifying these factors is crucial in determining the viability of recycling as a potential treatment for CFRPC waste. Therefore, there is a critical need for additional attention to techno-economic and life cycle assessment data and analyses of recycling CFRPCs, in particular mechanical techniques, given that any assessment relies heavily on data quality and availability. The deficiency in data regarding inputs and impacts poses a challenge in developing comprehensive life cycle assessments and techno-economic studies for CFRPC recycling. However, technical studies still suggest potential environmental benefits and opportunities for fiber reuse. While these advantages have not been fully demonstrated in a comprehensive life cycle study, they are recognized.

To provide an overview of a significant volume of CFRPC materials, it is worth noting that since the 1970s, these materials have been extensively utilized in various commercial products. For instance, half of the mass of aircraft comes from such materials. Some of the airplanes currently in production will reach the end of their operational lifespan. Consequently, a recycling process is essential to derive benefits from their waste materials, particularly CFRPCs. Lefeuvre et al. [26] assessed the aerospace sector’s worldwide CFRPC materials in both usage stocks and waste flow until 2050. They concluded that if the waste materials in this sector are not recycled, commercial aeronautics will create 500 K tons of accumulated CFRPC pollution by the end of this period. This will encourage more efforts needed to recycle and/or recover CF-reinforced composite materials. Likewise, Kooduvalli et al. [27] reported that approximately 8500 planes will be decommissioned by the end of the year 2050. This could potentially generate around 154,221 tons of CFs, which have the potential to be diverted from landfills and given a second life through recycling processes.

**Figure 3:**
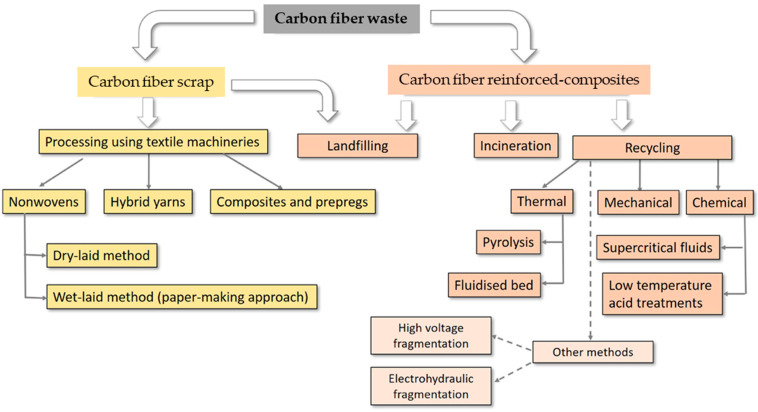
CFRPC waste and dry CF scrap management routes, adapted from [28].

Additionally, several aeronautical companies including Bombardier, Airbus, and EMBRAER widely use CFRPC composite materials, and subsequently the end of life of all these composites causes a large amount of waste. This waste encourages researchers to discover solutions to management and handling issues. Traditionally, many countries depend on both landfilling and incineration strategies for managing such waste. As mentioned earlier, these traditional strategies are not environmentally friendly due to gas emission and ground water pollution [25]. From a sustainable development and ecological protection perspective, a variety of methods can be adopted for managing CFRPC waste, including reuse, reduction, and recycling. Figure 3 shows the main strategies for managing CFRPC wastes and dry CF scrap.

Among the CFRPC management routes mentioned above, the recycling method is an effective one due to the conduciveness of its physical features and its suitable adaptation to management processes. CFRPCs can be recycled using three major techniques, including mechanical, thermal, and chemical processes, at both the academic and industrial levels [4,12,29,30]. Mechanical recycling causes fewer greenhouse gas emissions, which might improve environmental performance compared to other recycling techniques and even to traditional disposal procedures, i.e., landfilling and incineration [31,32,33,34].

**Figure 4:**
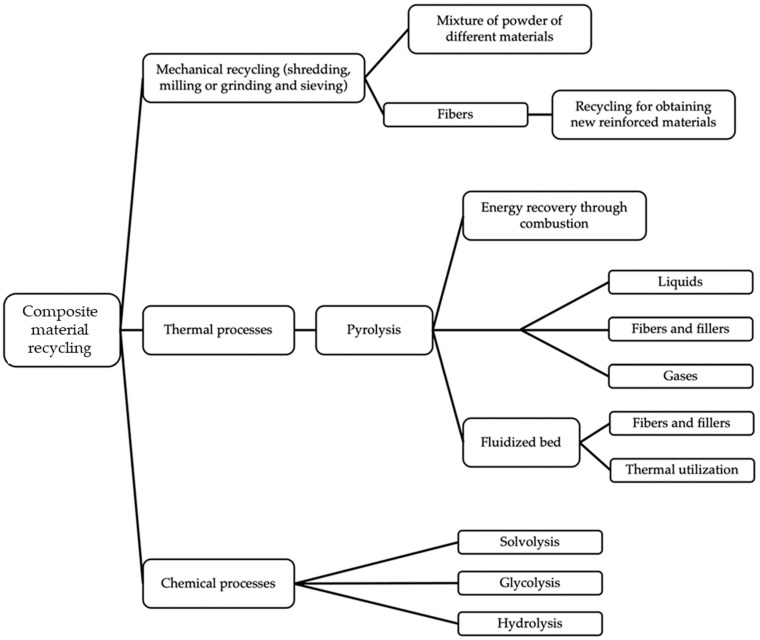
Classification of the three major recycling methods, adapted from [37].

While the environmental benefits are acknowledged, further investigation is still required to determine the suitability of the mechanical route for creating new, strong CFRPCs with isotropic properties. This is because of deterioration of the CF surface and shortening of their length during the milling, crushing, or grinding processes, which decrease the mechanical strength of CFs. As a result, there are presently few research and development activities focused on mechanical recycling to recover CF materials [34,35,36]. Figure 4 displays recycling approaches to CFRPC wastes, including the main techniques, sub-processes, and the products at the end of each technique’s process.

**Figure 5:**
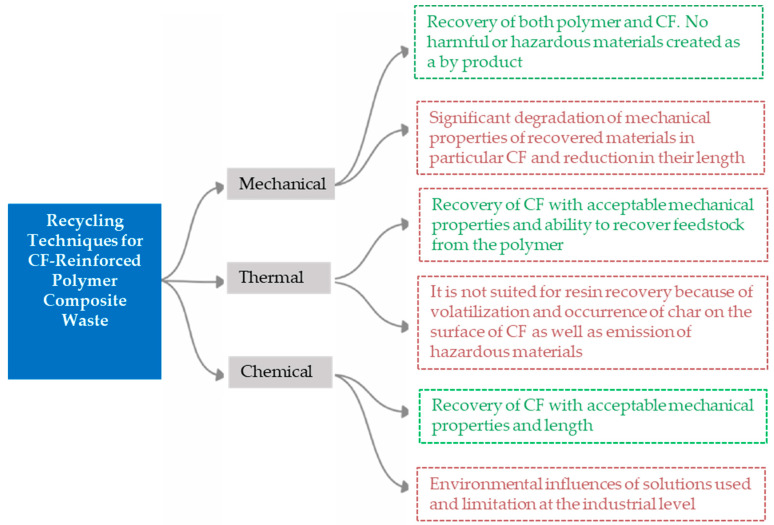
A comparison between the main recycling techniques for CFRPCs, adapted from [30].

Figure 5 illustrates a comparison between the three primary approaches to CFRPC recycling techniques, showcasing distinct outcomes in terms of fiber length, mechanical properties, and environmental impact. Thermal and chemical routes effectively separate fibers from the polymer matrix, contrasting with the mechanical technique, which typically yields chopped composite materials with significantly shorter CF lengths. Despite these differences, numerous institutes and companies around the world have embraced all these recycling and recovery methods [38]. In comparison to alternative methods, mechanical recycling of CFRPCs presents several advantages. First, it typically demands less energy input compared to high-temperature processes such as chemical and thermal recycling, contributing to reducing operational and manufacturing costs. The published literature indicates that the energy requirements for chemical and thermal recycling exceed those for mechanical recycling.

In this regard, Howarth et al. [39] proposed a model to analyze the energy needed for a mechanical recycling method for CFRPCs through a milling process. The energy was calculated and compared with the industrial scale-milling apparatus with a recycling rate of 10 kg per hour. The results indicate that the energy required for mechanical recycling is ~2.03–0.27 MJ per kg, which is significantly lower than the exemplified energy for virgin CF (i.e., ~183–286 MJ per kg). This suggests that substituting recycled CF for virgin CF is an effective means of reducing energy consumption. Such studies are significant for providing information and datasets for life cycle assessment and techno-economic results to evaluate the possible environmentally friendly benefits of utilizing recyclate materials (i.e., including all CFRPC materials that have been completely or partially recycled and serve as the basis for the manufacturing of new products).

In addition to the previously mentioned advantages, the simplicity of mechanical recycling procedures and the requirement of fewer chemical steps further support their attractiveness [39,40]. Additionally, its technology readiness level index often suggests that the mechanical recycling method is a viable alternative to other recycling processes [40,41]. Given that mechanical recycling avoids the use of high temperatures and/or chemicals to break down the polymer matrix, it emerges as the most promising approach for recovering carbon fibers (CFs) from CFRPC components. These attributes yield an added advantage to this process, as the recycled or recovered fibers and/or recyclate materials undergo no heating or chemical alteration. It can be applied to the majority of industrial fiber typologies without limitations. The swift industrial scalability, coupled with environmental and economic sustainability in terms of specific energy demand, are compelling features of mechanical recycling that increase its attractiveness [42]. Table 1 provides a summary of the energy consumption rates, environmental impacts, and technology readiness levels of mechanical, chemical, and thermal recycling techniques.

In this context, Asmatulu et al.’s review [44] of CFRPC recycling technologies outlines energy requirements, discusses product outputs, and examines the quality of recyclate materials, emphasizing the potential for sustainable CFRPC manufacturing. Their conclusion, within a life cycle analysis framework, underscores the various benefits associated with exploring the end-of-service life of composite products, spanning environmental, educational, health, social, and economic domains. Consequently, mechanical recycling has been selected as the focus method in the current review. While numerous published studies provide a general overview of recycling techniques for CFRPCs, our knowledge indicates a lack of a single study exclusively covering the mechanical recycling approach.

This gap arises from the lack of extensive experimental studies on chemical and thermal techniques compared to the mechanical method. Challenges in obtaining long and robust CFs through mechanical recycling may contribute to this disparity. Nonetheless, mechanical recycling remains crucial for recovering short and chopped CFs with suitable mechanical and electrical properties, which are applicable for new CFRPC products that can be used in several industrial applications. This highlights a research gap in the review of the mechanical recycling technique, specifically in comparing the performance and properties between experimental and simulated data (i.e., numerically predicted damage behaviors and characteristics) for mechanically recycled CFRPCs and their counterparts in new or virgin CFRPCs. Consequently, this review aims to fill this gap by providing a comprehensive understanding of existing and developed mechanical recycling approaches for CFRPCs, along with simulation models of recycled CFRPCs. The scope encompasses current and potential applications, as well as associated limitations and challenges, recognizing that the recyclability of such composite materials is vital for their future success.

## 2. Mechanical Recycling of CFRPCs

**Figure 6:**
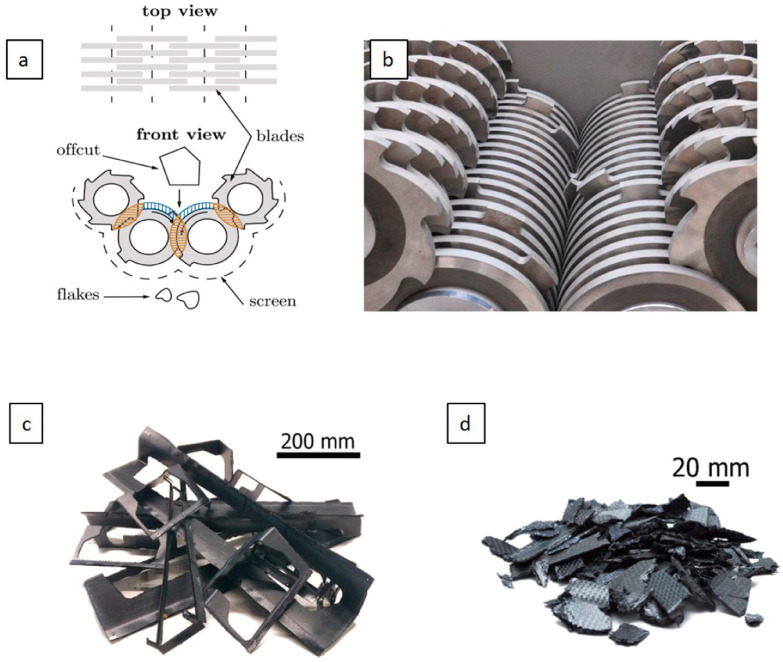
Illustration of multiple shaft machine: (**a**) front and top views, (**b**) inside view, (**c**) CFRPCs before shredding, and (**d**) CFRPCs after shredding, adapted from [45].

The first technique used for recycling CFRPC materials is based on the mechanical recycling procedure. This procedure relies on crushing, shredding, and milling of the CFRPC parts into smaller pieces that can then be additionally ground into a fine powder. This powder is usually utilized for polymeric filler applications that are perhaps feasible for creating new products, therefore avoiding landfilling and incineration. Outputs from mechanical recycling are classified into three categories, namely short CFs, fine powders (i.e., a combination of resin and fibers with a higher resin content), and coarse recyclate materials (i.e., a mixture of resin and fibers with a higher fiber content). The different sizes of recyclate products allow them to be recovered and isolated using cyclones and sieving processes. To perform this mechanical approach, equipment such as multiple shafts is commonly used, as can be seen in Figure 6. The shredding products are large and identical pieces of around 50–100 mm (about 3.94 in) in size, controlled by the distance between blades, the sieve size, and their rotation speeds [45,46,47]. These products can then be ground into both micro- and nano-size powders.

**Figure 7:**
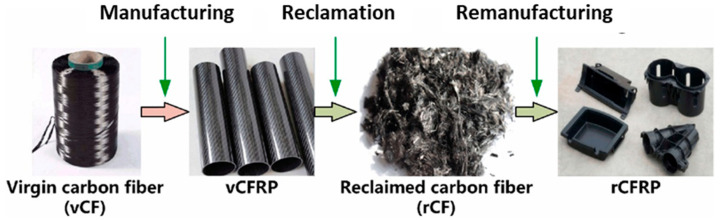
The stages of the recycling processes, modified from [47].

Accordingly, recycling of CFRPCs could be divided into two stages, including reclamation of CF and then fabrication of a new micro- and nano-composite material, as shown in Figure 7. Vincent et al. [45] developed a new method that calculates fiber length distribution for flakes that are produced from shredded CFRPC samples. They reported a strong relationship between shredding machine parameters and CF length distribution into the developed CFRPCs.

Additionally, Pimenta et al. [48] reviewed market feasibility of the recycling of CFRPC scrap for structural applications. They concluded that some recycling and remanufacturing processes are at a mature stage, with executions at commercial scales. The main technical challenges relate to waste management and quality control of recycled products. Researchers have explored mechanical recycling techniques and their effect on the mechanical properties of CFRPCs and feasibility in terms of performance, environmental, and economic aspects.

**Figure 8:**
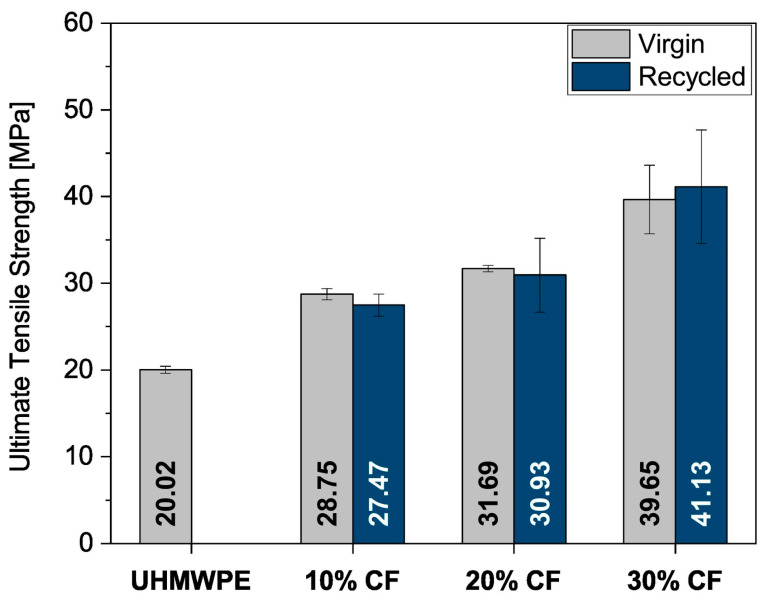
Tensile strength of CFRPCs reinforced with virgin and recycled CFs, adapted from [20].

For example, Ventura et al. [20] compared the mechanical properties of ultra-high-molecular-weight polyethylene composites reinforced with both virgin and recycled CFs. Their results show that the composite containing recycled CF displays a comparable ultimate tensile strength to its virgin counterpart, as shown in Figure 8. A similar conclusion has been reported by Tomioka et al. [14] for CF-reinforced polypropylene composite materials. These findings enable and encourage the use of recycled CFs instead of the use of virgin CFs in several applications.

Furthermore, Li et al. [49] studied the economic and environmental feasibility of mechanical reclamation of CF composite waste. They revealed that using recycled CF to relocate virgin CF might considerably improve environmental and economic performance. They reported that mechanical recycling is the only route that can reduce energy and gas emissions with a low amount of landfilling waste. However, they concluded that this technique is only feasible if recycled CF replaces virgin CF on a large scale to increase revenue.

It has been reported that a kilogram of recycled short CF could reduce both cost and energy consumption by 55% and 89%, respectively [20]. In addition, Palmer et al. [50] reported that mechanical recycling could be improved through careful attention to separation and reformulation practices. The authors stated that these factors affect the economic feasibility of the regrinding process as a potential solution to the CFRPC recycling problem. A similar study investigating the mechanical recycling of CFRPCs by peripheral down milling was carried out by Durante et al. [43], who highlighted that the peripheral down-milling technique can exemplify a viable method for obtaining economic and environmental advantages.

Song et al. [51] studied the mechanical recycling process of CFRPCs with a ball-milling machine. The obtained recyclate fibers were used as reinforcement agents without any further treatment and separated from the polymer matrix. The authors reported that the obtained fibers were in powder form with an average CF length of ~59 μm. There was about 98% and 75% enhancement in specific compressive and tensile strength, respectively, of the developed new CFRPCs, with excellent interfacial bonding between the recycled CF and the polymer matrix (i.e., epoxy foam) for up to 10% by weight of the attained recyclate fibers. Another study was carried out by Ogi at al. [52], who ground CFRPC pieces with a ball-milling machine. Most of the obtained pieces were in the particle form with sizes of 1 to 10 µm, including many separated fibers and large-sized bundles. Acrylonitrile butadiene–styrene was mixed with the obtained pieces to develop new CFRPC materials. The strength and modulus of the developed composites increased considerably, with an increase in recyclate fiber loading of up to 50 wt.%.

A similar study was carried out by Thomas et al. [53], who obtained a carbon powder with an average size > 1.25 mm (about 0.05 in) from the process of cutting and grinding laminate CFRPC waste followed by the sieving approach to remove large particles that could affect the compounding method for the new CFRPC materials. The obtained powder was incorporated into epoxy resin to enhance the mechanical features. The results showed an improvement in some properties of the developed composites, including their tensile, flexural, and impact strengths. Mamanpush et al. [54] conducted a similar study that developed recycled CF-reinforced polyether ether ketone composite materials. The recycled CF was obtained from epoxy/CF and vinyl ester composite wastes. A shredder followed by a hammer for milling were used to reduce the final particle size to <3.4 mm (about 0.13 in) mechanically refined these wastes. The results show that adding both recyclate fibers to the composite system enhanced the flexural properties of the final composites.

A comparable study was undertaken by Li et al. [55] on recycling aerospace industry waste made of CFRPC products using a milling technique followed by shredding. The resulting recyclate materials were compression molded into flat pallets and subjected to mechanical testing, revealing a minimum 50–60% decrease in mechanical properties compared to the original CFRPCs. Nevertheless, the authors noted that as the particle size of recyclate materials decreases, the mechanical properties of the newly developed CFRPC increase.

Al-Zahmi et al. [47] fabricated CFRPC materials from a recycled polylactic acid matrix reinforced with prepreg and CF sheets. These sheets are firstly shredded into small pieces using a shredding machine before the incorporation with the polylactic acid matrix. The study concluded that the developed composites demonstrated properties that could be used for several applications. Takahashi et al. [56] crushed CFRPC waste into squares of about 1 cm (about 0.39 in). Then, new CFRTP materials were developed with the crushed recyclate fibers using thermoplastics (i.e., acrylonitrile and polypropylene). Their results showed that the strength and modulus of both composite materials containing recycled fiber and virgin fiber were almost the same up to 24% loading. Further incorporation of CF materials into the polymer matrix induced a reduction in such properties. This may be caused by the poor impregnation of thermoplastics into recycled CF.

A similar conclusion has been drawn by Colucci et al. [57], who considered the impact of the mechanical recovering technique on the mechanical properties of polyamide reinforced with 30% by weight of short CFs. The authors concluded that the recycling technique did not affect the investigated properties significantly. Despite some decrease in the average length of fibers occurring during the grinding stage, a negligible decrease in tensile strength was observed. The authors also concluded that the mechanical recycling approach could promote the effective recycling of CFRTP in automotive industries. Kiss et al. [58] produced composite panels from CFRPC waste materials. These panels were shredded to fine and coarse portions. Sieves of 4 mm size (about 0.16 in) and 8 mm (about 0.31 in) in diameter were utilized during the shredding processes. Panels were shredded into 0.5–7 mm in fiber length (fine fraction) and 5–25 mm (coarse fraction). The shredded pieces displayed inadequate properties for high-performance applications. This behavior could be due to the random fiber orientation after the shredding processes. However, injection molding resulted in strong flow-induced fiber alignment, and consequently, significantly better properties were observed. Hence, the type of processing for fabricating new CFRPCs is a very important factor to be considered during their life cycle assessment.

To summarize this section, the chemical approach yields the most robust recycled CFs compared to their virgin counterparts, followed by the mechanical recycling technique. Mechanical recycling emerges as the most viable method, and is widely practiced in both academic and commercial sectors. The substitution of mechanically recycled CFs for virgin ones has the potential to significantly enhance both the environmental and economic aspects of recycling. However, a potential reduction in the mechanical properties of recycled CFs remains a theoretical consideration. Nevertheless, all waste materials containing CF elements can undergo mechanical treatment and be repurposed as fillers and reinforcements in a new composite material system tailored for specific applications.

## 3. Finite Element Modeling of Recycled CFRPCs

**Figure 9:**
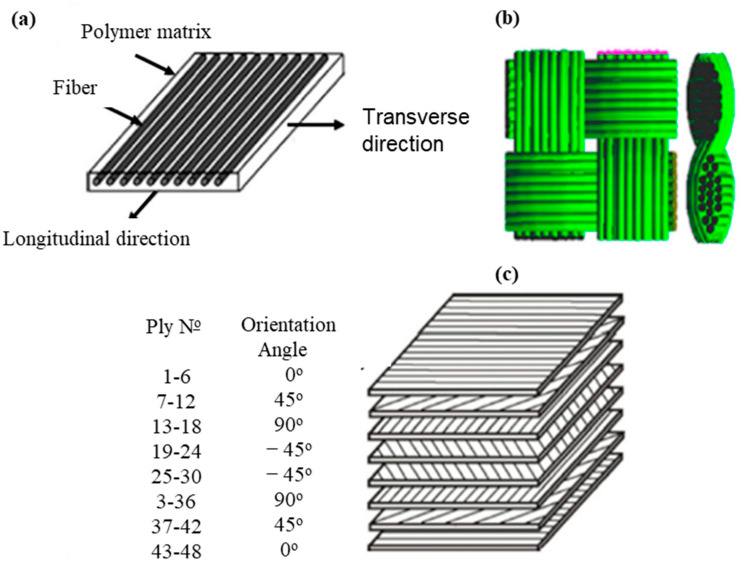
Scheme of CFRPC structures: (**a**) unidirectional CF orientation, (**b**) bidirectional CF orientation (woven), and (**c**) multi-CF orientation laminate, quasi-isotropic laying-up sequence [0°/45°/90°/−45°]. Modified from [59].

Though there are increased applications of CFRPCs in numerous industries, producing such materials with isotropic properties and predicting their life cycle are still challenges due to their anisotropic and heterogeneous nature. Anisotropic materials display different mechanical properties in each direction; i.e., they are not uniform with respect to all planes and/or axes. This behavior is anisotropic; because of this, the CF can be either unidirectional (Figure 9a) or bidirectional (Figure 9b). In addition, unidirectional CF can be aligned on a thin plate, pre-impregnated with polymer to define the stacking order and layer for composite laminates, as can be seen in Figure 9c.

These structures are most typically used for manufacturing CFRPCs on the industrial scale. The orientation of each layer of composite laminates and their stacking order can be controlled to develop a wide range of mechanical properties and performances of the final CFRPC products [60,61]. Several modeling techniques have been proposed by some researchers to predict the mechanical performance and the lifetime of CFRPCs during the design stages before manufacturing the final products of such composite materials. Likewise, finite element analysis (FEA) is one of the most common techniques in terms of numerical solutions that have been applied to address these challenges. FEA methods have been broadly utilized as numerical tools for analyzing and anticipating the mechanical properties and performance of engineering materials involving CFRPCs. There are many sectors that benefit from composite material simulation, such as the aerospace, aeronautics, energy, automotive, civil, sports, and even electronics sectors [59,62,63,64,65,66]. This is because FEA is a great method to prove designs before an objective prototype of the CFRPC is fabricated. Nevertheless, it is possible to understand and anticipate many phenomena, including damage and the CF and matrix interface, when the CFRPC is exposed to severe conditions, such as dynamic loading, high temperatures, and high pressures, when using the FEA approach [59,62,63].

Moreover, modeling the CFRPCs involves going through three levels: the microscale, mesoscale, and macroscale. The microscale focuses on the CFRPCs’ attitude, where the interactions from the essential components of the materials are thoroughly inspected considering their heterogeneous nature. The macroscale studies the behavior of composite materials assuming homogeneity, with the effects of all constituent materials detected solely through the composite material’s average apparent properties. The mesoscale is an ideal choice, as the nanoscale has been rising significantly according to the growth in nanomaterials as reinforcements as well as the necessity of comprehending damage sequences [59,67].

Numerous studies, encompassing both numerical and experimental approaches, have extensively explored various properties and performance aspects of virgin CFRPC materials. These investigations predominantly focus on the unidirectional structure [67,68] and bidirectional (woven) structure [69,70,71] of CFRPC materials. However, there is a limited emphasis on studies comparing both experimental and simulated data for mechanically recycled CFRPCs in comparison to new CFRPCs. This research gap could be attributed to the complex process involved in the random distribution of CFs, considering their short lengths, sizes, and orientations. Furthermore, the utilization of low-quality recyclate materials serving as trivial fillers may contribute to the existence of this research gap.

The significance of integrating mechanical recycling with FEA methodology lies in its pivotal role within the circular economy. FEA addresses the imperative for sustainable practices by confronting challenges and leveraging opportunities in CFRPC recycling. Given the substantial predicted CF waste (see Figure 1), both experimental and numerical approaches are urgently needed to investigate the properties and failure mechanisms of such CF-reinforced composite materials. Therefore, the incorporation of FEA models becomes crucial, facilitating a streamlined design process and comprehensive material assessment. This integrated approach not only promotes sustainable practices but also enhances resource efficiency, reduces waste, and optimizes methods [72,73].

To assess the effective properties of short CF-reinforced polymer composites, a representative volume element algorithm based on the FEA technique has been proposed. For instance, Yan et al. [74] propose an efficient recycling approach for retired wind turbine blades, introducing a modified representative volume element: a minimum volume sample yielding representative values for the entire CFRPC. Widely accepted for simulating the short random distribution of CFs, their strategic approach aims to reduce wind blade waste costs while preserving the mechanical properties of recycled blade samples. This proves invaluable for predicting the properties of 3D-printed materials derived from recycled wind turbine components.

Numerous studies have been conducted to analyze the mechanical properties of recycled CF-reinforced epoxy composites using FEA. For instance, Ping Liu et al. [75], Magino et al. [76], Guo et al. [77], and Tang et al. [78] introduced finite element techniques to create effective geometrical models of CFRPCs using short and/or chopped CF-reinforced polymer. These proposed models can serve as a baseline when designing CFRPCs using recycled CF. Bai et al. [79] determined the representative volume element for CFRPCs and it is valid in terms of accuracy and the actual effect of different CF arrangements within the CFRPC constituents. Similarly, Wang et al. [80] considered the volume fraction, aspect ratio, and packing geometry of CF through the proposed representative volume model to simulate the viscoelastic behavior of short CF-reinforced polymer composites. The results show that adding more CF to a material makes the whole composite less compliant mechanically, and the CF reinforcement’s impacts in the direction of CF alignment will be more pronounced. The model can speed up the analysis of elastic and viscous behaviors for a complex finite element material that contains short fiber-reinforced parts.

Another representative volume model-based modeling approach is proposed by Tang et al. [81] to predict before-and-after failure responses of chopped CF considering distinct loading cases and complex stress states. They evaluated and calibrated their model by uniaxial tensile, compressive, and in-plane shear experimental activities. They found that the developed model can be used to predict failure envelopes under complex conditions. Moreover, Gopalraj et al. [68] developed an FEA methodology to investigate the damage behavior and mechanical properties, mainly tensile and impact strengths, of recycled CF reinforced with epoxy resin. Both experimentally and numerically determined, the results display that the adopted FEA model can validate the experimental results in terms of the mechanical properties and defect behavior of investigated CFRPCs that have inconsistent and complex performance, geometry, composition materials, and mechanical behaviors. Choi et al. [82] investigated the failure of a hybrid of short and continuous CF-reinforced polyamide composites. They confirmed the reliability of the proposed model with a certain correlation coefficient and they concluded that the developed model can predict the fatigue performance of CFRPCs, considering both multi-axial stress and anisotropy states. Furthermore, Tarkar et al. [83] concluded that the simulation data for polymers reinforced with short CF would further support structural applications of complex CFRPC components.

Equally, in order to enhance the mechanical features in 3D printing, it is necessary to comprehend the structure–property relationship of CFRPCs. Accordingly; Tang et al. [84] introduced a new model to estimate the mechanical attitude of CFRPCs using the representative volume element algorithm. A short CF was mixed inside the CFRPCs in this study; its distribution constantly plays a key role in mechanical performance (the mechanical performance is constantly affected by how the fiber is distributed). A 3D fiber orientation tensor was employed in the microscale to describe the fiber’s spatial distribution. They found that the mechanical performance of CFRPCs could be improved by increasing the fiber volume fraction and the alignment of fibers along the loading direction. The developed model in their study was sufficient for characterizing the mechanical properties of the produced CFRPCs.

Somireddy et al. [85] who predicted the mechanical behavior of CFRPCs that were also produced using a 3D printing technique, have carried out a similar study. The authors suggested using the laminate theory that precisely estimated the mechanical behavior of 3D-printed parts of short CF-reinforced composites. They concluded that laminate theories could be used in the initial design and analysis of printed laminates to improve the printed parts’ mechanical performance. Moreover, the 3D parts’ performance and printing quality are influenced by several considerations, such as infill density, infill pattern, layer resolution, print speed, raster angle, bead shape, printing temperature, print-bed temperature, build orientation, and so on. Consequently, Al Rashid and his colleague [86] developed a model for the thermomechanical performance of 3D-printed chopped CF-reinforced polyamide-6 composites using a numerical simulation tool to predict how the 3D process induced deflections, residual stresses, and warpage in 3D-printed specimens. A significant impact of infill pattern and density is observed on deflections, residual stresses, and warpages from the numerical simulation results.

Considering the literature reviewed above, there remains a research gap concerning the comparison of performance and properties between experimental and simulated data for mechanically recycled CFRPCs and their corresponding new or virgin CFRPCs. The limited research in this field can be attributed to various challenges. The variability in fiber length and orientation resulting from mechanical recycling presents a barrier in accurately simulating the material’s behavior. Shortened and randomly oriented fibers introduce complexity to models, making it challenging to understand the intricacies of fiber damage, matrix residual stresses, and their collective impact. The lack of standardized material properties for recycled CFRPCs impedes the development of reliable FEA models, as accurate input data are crucial for simulation fidelity.

Capturing the effects of recycling-induced alterations is challenging due to limited knowledge about the interactions between recycled CF and other materials in practical applications. The absence of comprehensive understanding of recycling-induced changes in CFRPC microstructure and predicting fatigue behavior further adds complexity to simulations. Scaling up FEA models to larger structures faces obstacles due to recycling-induced heterogeneity, emphasizing the need for scalable modeling approaches that consider varying material properties.

Additionally, the environmental impact of mechanical recycling processes is often excluded from simulations, indicating a critical gap in holistic assessments. The validation of FEA models for recycled CF requires comprehensive experimental datasets, underscoring the interdisciplinary nature of addressing these challenges and the importance of aligning simulation results with real-world behavior. Addressing these challenges necessitates concerted efforts in material characterization, experimental validation, and improving modeling techniques, establishing a cohesive foundation for advancing the sustainable application of recycled CF in engineering designs.

To summarize this section, the progress in FEA methodologies streamlines the design process for prototypes or products featuring intricate geometries made of short and/or chopped recycled CF-reinforced composite materials. This approach minimizes the trial-and-error impact, enabling designers and researchers to accurately predict fatigue life. The majority of experimental and numerical studies conducted validate that the adopted FEA model aligns with the respective experimental results for mechanical properties and defect behavior in investigated recycled CFRPCs, which exhibit inconsistent and complex performance, geometry, composition materials, and mechanical behaviors.

## 4. Limitations of Mechanical Recycling

Mechanical recyclability is affected by the mechanical properties of CFRPCs. Both researchers and developers have shown a great deal of awareness of recycled CFRPCs due to their remarkable and wide range of both mechanical and electrical properties and the potential of exploiting them in several industrial applications.

Additionally, these properties can be modified or enhanced by selecting appropriate raw materials, understanding their characteristics, and implementing recycling methods. For instance, considerations such as the length and alignment of CFs within the polymer matrix, as well as whether their surfaces have been treated and modified, play significant roles. Any adjustment made during the recycling and manufacturing processes impacts the properties and performance of resulting CFRPC products, thus influencing their suitability for specific industries. Certain properties, such as the tensile strength of CFRTP in mechanical applications, are particularly crucial. Furthermore, the reduction in mechanical properties may occur due to deterioration of CF surfaces and shortening of fiber length during milling, crushing, or grinding processes, leading to decreased tensile strength in recycled CFRPC materials. Previous studies [6,7,8,9,21,45,51,52,53,54,55,56,57] have examined the effect of fiber length and orientation on composite product performance, with findings suggesting a strong correlation between fiber length and final composite material performance. This underscores the importance of fiber length in influencing stress transfer capabilities, particularly when comparing longer fibers.

Nevertheless, the reduction in CF length had a negligible impact on the mechanical properties of some recycled composites, which revealed the significance of using recycled CF as a reinforcement agent instead of virgin CF in producing composite material products [20,47,50,51,52,53,56,57]. In addition to these findings, mechanical recycling of CFRPCs is essentially limited because of their complex composition (heterogeneity) and the cross-linked nature of the resin (recyclate materials), quality of the products, and subsequent lack of a recognized market. Moreover, the role of some other factors such as CF dispersion, distribution, orientation, and arrangement within the new polymeric matrix and the optimized processing molding procedure should be examined comprehensively for developing new CFRPCs using recycled CF or recyclate ingredients. These factors influence the adhesion (interfacial properties) between the CF and the polymer matrix.

**Figure 10:**
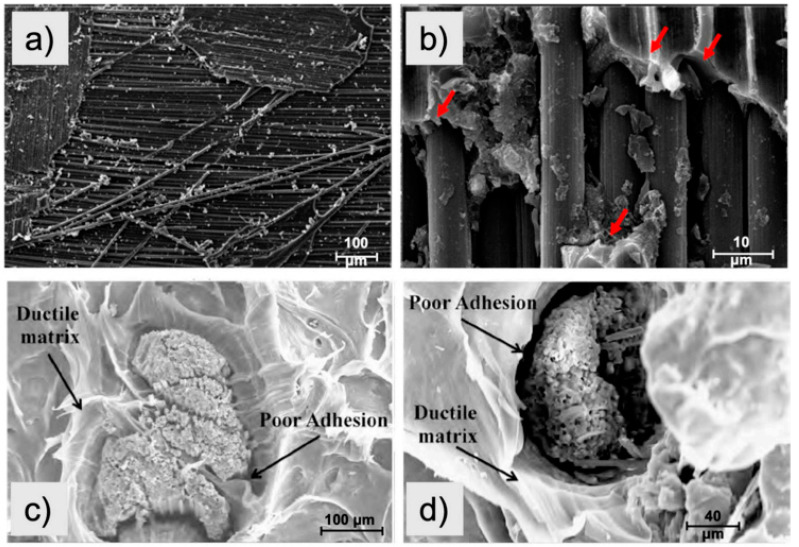
SEM images of the recycled CF: (**a**,**b**) poor interfacial adhesion of recycled CF-reinforced epoxy matrix; (**c**,**d**) poor interfacial adhesion between the polypropylene matrix and recycled CF, adapted from [37]; red arrows shows segregation the separation between the epoxy matrix and recycled CF.

Consequently, one of the main problems with CF being mechanically recycled is the adhesion between it and the new polymeric matrix, in particular both thermoset and thermoplastic polymers. Figure 10 shows an example of this main issue. For example, Figure 10a,b display SEM images of an interface between thermoset resin (i.e., epoxy) and recycled CF at different magnifications while Figure 10c,d show fracture surface images of a thermoplastic resin (i.e., polypropylene) reinforced by a recycled CF. It is clear that there is poor interfacial adhesion in both examples. Thus, the recycled CF–polymer matrix’s adhesion is a critical factor that needs to be addressed before reaping the full potential of CF. In order to resolve this challenge, modification of the CF surface is usually needed.

Therefore, a noteworthy research challenge in the mechanical recycling of CFRPCs focuses on improving the adhesion between recycled CFs and new polymer matrices. Several studies have concentrated on enhancing adhesion through surface modification techniques or through alignment of CFs. Among these enhancements, plasma treatment [87] stands out by exposing the CF surface to reactive species, heightening reactivity, and fostering improved adhesion with polymer matrices. This plasma technology alters the surface chemistry of CF, generating functional groups that enhance compatibility. For example, Lee et al. [88] examined how the plasma surface treatment influenced the flexural strength and adhesion qualities of recycled CF when they reinforced a new polymer matrix. They discovered that plasma processing is quicker and less expensive than traditional oxidation methods, showing an increase in flexural strength by over 17% compared with that prior to the plasma treatment. In addition, the authors found that the O/C level after treatment was about two times higher than that before treatment (i.e., it increased from 11% to 25%). This can be attributed to the introduction of oxygen-containing groups on the surface of recycled CFs.

Additionally, chemical grafting has been utilized to modify the CF surface by attaching specific functional groups. Likewise, coupling agents play a pivotal role in improving adhesion between recycled CF and the polymer matrix in composite materials. For example, both acids and silane compounds used in alignment of CFs, widely used as coupling agents in CF-reinforced composites, form chemical bridges between the CF surface and the polymer matrix. These processes enhance adhesion, promote load transfer between the two components, and allow them to form a strong covalent bond [89,90,91,92]. For instance, Lee et al. [90] explored the impact of silane coupling agents’ chemical properties on the interfacial bonding strength between thermoplastic polymers (i.e., polyamide 6) and recycled CFs. This coupling agent introduced new chemical groups (i.e., amino, carbonyl, and hydroxyl) on the surface of the CFs. The most effective results were achieved using a 1 wt.% concentration for a 10 s treatment, resulting in enhancement in the interfacial bonding strength to 32 MPa, approximately 15% higher than that of commercial CFs in a polyamide 6-based application. Generally, they found that the interfacial shear strength characteristics are comparable to those of commercial CFs, which are typically coated with epoxy sizing agents. This outcome is attributed to the resizing of the CFs in their study, achieved by selecting coupling agents that had excellent chemical coordination with each resin after surface treatment with nitric acid. A similar study has been carried out by Zhu et al. [91], who found that the addition of a suitable amount of the silane agent (<4 wt.%) can significantly improve the mechanical properties of recycled CF-reinforced bio-based epoxy composites. This improvement can be attributed to the cross-linking and curing reaction between the amino groups of the silane and the epoxy matrix that improved the interfacial bonding. Thus, an appropriate concentration of the silane coupling agent is critical to improve the mechanical properties of recycled carbon fiber-reinforced bio-based epoxy composites.

In addition, incorporating and/or growing nanomaterials directly, such as carbon nanotubes (CNTs) [93], into the polymer matrix alongside recycled CFs can enhance overall adhesion properties (the fiber/matrix interface adhesive bond) and result in enhanced mechanical properties while retaining a smaller delamination area. Nanomaterials serve as additional reinforcement, improving the interfacial interaction between CFs and the polymer. For instance, it has been reported that utilizing patterned growth of CNTs on the surface of CFs by the modified catalytic chemical vapor deposition method improved the strength and impact energy dissipation of the CFRPCs by 11% and 127%, respectively [94]. In comparable studies [95,96], the flame-synthesized technique was used as an energy-saving method to graft functional CNTs onto CFs. Similar conclusions were drawn, as enhanced adhesions of the flame-grown CNTs to CFs were realized. Moreover, Fazeli et al. [97] improved the mechanical and interfacial properties of recycled carbon fibers by co-functionalizing them with CNTs through an electrophoretic deposition method. The results indicated that the CFRPCs functionalized and recycled by CNTs exhibited substantial enhancements in their tensile and flexural strengths, with increases of up to 32% and 27%, respectively, compared to unmodified counterpart composites.

The aforementioned approaches enhance interfacial adhesion and the overall mechanical properties of recycled CFRPC materials. Nevertheless, their effectiveness depends on diverse factors such as the type and size of CFs, their arrangement within the polymer matrix, the type of polymer matrix, and the processing techniques utilized in developing CFRPC products. Additionally, it is crucial to ensure that the enhanced adhesion achieved through surface modification remains stable over time, particularly under diverse environmental conditions, before fully realizing the potential advantages of any modification. Therefore, thoroughly studying and validating the long-term performance (durability) of recycled CFRPCs is essential.

It is worth mentioning that some surface modification techniques, particularly those involving advanced chemical treatments, may be cost-prohibitive for large-scale industrial applications. Hence, developing cost-effective methods that can be scaled up without compromising modification quality is significant for the mechanical utilization of recycled CFs. Therefore, end-of-life considerations, including understanding the impact of surface modifications on the recyclability of CFRPCs, are also crucial issues, as some modifications may introduce elements that complicate the recycling process or reduce material recyclability. Lastly, the lack of standardized testing and protocols for evaluating the effectiveness of surface modifications complicates comparisons between different studies. Establishing universally accepted standards and procedures for assessing adhesion improvement is also vital.

To summarize this section, the recycled CFRPCs exhibit significant potential for various industrial applications. However, current activities and efforts predominantly remain at the research level. Specifically, there is a focus on enhancing the adhesion properties of newly developed CF-reinforced composites utilizing recycled CF or recyclate materials.

## 5. Current and Potential Applications of CFRPCs

**Figure 11:**
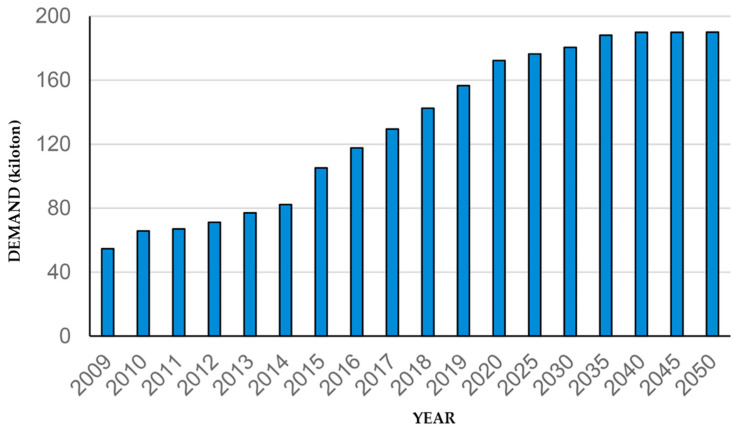
Global CFRPC demand in kilotons by year, adapted from [13].

Many companies have already adopted CFRPC products across a variety of sectors in the world. Due to their exceptional properties and performance, they are ideally suited for a wide range of applications. Figure 11 shows the worldwide demand for CFRPCs. This demand is anticipated to grow to ~190 kilotons in 2050, which is relatively comparable with the anticipation of the global demand for CF and the evaluation of its waste arising from the composite materials industry (see Figure 1).

For instance, CFRPCs are used in oil and gas applications for pipelines instead of metallic materials, i.e., steel, to avoid the corrosion effect when corrosive materials are transported, which has led to a reduction in maintenance costs. Typically, the damaged metallic pipelines are removed totally and substituted with new ones or are protected with an external sleeve as reported by Saeed et al. [98]. For illustration, the CF-reinforced polyurethane matrix is one of the typical composite materials used to design composite overwrap repairs for pipe pressure issues. Yu et al. [99] reported that pipes made of CFRPCs have been recognized as a vital substitute for pipelines made of metallic materials due to the diverse advantages of these non-metallic materials, such as a high stiffness-to-weight ratio, improved fatigue resistance, and improved corrosion resistance. Venkatesan et al. [100] considered the mechanical properties of CFRPC materials in deep-sea applications. The authors concluded that the examined properties were not impacted by the sea environment or degradation or corrosion, and formation of biofilm was not witnessed. A review article by Guo et al. [101] provided the most relevant information on the fatigue behaviors of CFRPCs. Generally, fatigue loading is the main parameter leading to the performance degradation of CFRPCs that adversely disturbs their damage tolerance and service life. Moreover, use of CFRPC products is growing in both airplanes and automobiles, which could lead to a drop in greenhouse gas emissions effects, decreases in weight (40–50% weight reduction in comparison with steel products [101,102]), and subsequent reductions in energy [103,104]. For instance, manufacturers have been manufacturing CFRPC materials for automotive body panel parts, and there is the potential for structural and nonstructural part applications [48,103,105,106,107,108]. Another example of using CFRPCs in the automobile industry is creating a prototype of an automotive body in order to enhance fuel efficiency and sustainability. This body was prepared entirely of recyclable materials (20 wt.% recycled CF and 80 wt.% recycled polyamide), as reported by Faruk et al. [109] and their colleagues. Kiss et al. [58] and Friedrich et al. [103] revealed that recycled CFRPCs can be used for future opportunities in automotive applications. In addition, Colucci et al. [46] concluded that the recycled CF-reinforced polyamide matrix could be effectively used for structural and/or semi-structural automotive purposes, promising decent final performances and benefits from the environmental point of view. Souppez et al. [110] provided an innovative quantitative valuation of recycled CF for automotive industrial parts, which might contribute to future developments in sustainable CFRPCs in the automotive industry.

In terms of potential renewable energy applications of CFRPC products, both Thomas et al. [111] and Murray et al. [112] explained the advantages and disadvantages of such composite materials for wind turbine blade applications. Furthermore, Garate et al. [113] developed a recyclable CFRPC for wind turbine blade materials. The rapid development of the wind energy sector, with a steady increase in wind turbine size, has led to increasing consumption of the CF used in wind turbine blades [114]. An overview of the recycling and reuse of wind turbine blades has been presented, along with the scientific data available for different types of materials as well as recycling processes, by Jani et al. [115].

In building and construction applications, recycled CFRPCs have been incorporated into cementitious composites with an enhancement in the workability of cementitious materials up to a certain load of recycled CF [116]. The authors concluded that the addition of recycled CFRPCs encourages sustainable development of materials; however, it exposes challenges related to the recycling of CFRPCs, and the properties of these recycled materials, in particular their durability properties, need further investigation. Yuan et al. [117] investigated the feasibility of combined use of these recycled CFRPCs with rubberized concrete. They concluded that recycled CFRPCs are promising materials for consumption in civil infrastructures in the building and construction sector. Overall, the application of CFRPCs can not only improve the mechanical properties of certain products for specific applications, but is also of great advantage to the ecological environment. Ultimately, creating new markets for recycled CFRPCs necessitates the establishment of an economically sustainable recycling model, grounded in comprehensive life cycle assessments and high-quality techno-economic data.

## 6. Conclusions

There has been a notable increase in interest in sustainable materials due to environmental concerns caused by the waste from various polymeric matrices and their composites. As a result, this review provides a comprehensive overview of carbon fiber-reinforced polymer composites (CFRPCs), carbon fiber (CF), and the recycling of their waste materials. This is because the recovery and reuse of such materials are crucial activities for future applications due to their exceptional properties and features. Additionally, recycling end-of-life CFRPC products can reduce environmental impact, enhance sustainability, and help create a circular economy by reprocessing waste into value-added materials. This review also provides information on the life cycle assessment and global demand for CF and its waste.

There are three major techniques for recovering CF, including mechanical, thermal, and chemical methods. While numerous published studies address the recycling techniques for CFRPCs and provide a general overview of such techniques, to the best of our knowledge, there are currently no reviews covering the mechanical method in detail. Therefore, this review focuses on the research and development activities related to the mechanical method. Mechanical recycling of CFRPCs has proven to be an effective method of recovering valuable materials, reducing waste, and avoiding landfilling and incineration.

Finite element analysis (FEA) has emerged as a commonly used technique for CFRPC simulation, which can predict a wide range of phenomena and properties. Continued research on both experimental and simulation activities will undoubtedly lead to further improvements in their properties and applications in various industries. While research and development, including FEA, to evaluate virgin CFRPC materials have been well investigated and established, there are no significant studies involving recycled CFRPC materials. How to carry out FEA for recycled waste materials is a crucial issue to be solved in follow-up research. Thus, in the present review, FEA modeling of some recycled CFRPCs has been reviewed to fully understand the advantages and challenges of predicting the properties and performance of such composite materials.

Finally, current applications of recycled CFRPCs and the limitations of the mechanical recycling method are stated in this review. This review highlights the evident need for additional focus on techno-economic aspects, life cycle assessment data, and analyses of CFRPC recycling. The findings underscore that composite recycling is in its early stages, emphasizing the necessity for further study and in-depth research.

## Figures and Tables

**Table 1:** Energy consumption rates, environmental impacts, and technology readiness levels of mechanical, chemical, and thermal recycling techniques.

Recycling Technique	Processing Temperature/Pressure [43]	Energy Consumption [39,43]	Environmental Impact [43]	Technology Readiness Level [40]
Mechanical (crushing, shredding, and milling)	Room temp/Atmospheric pressure	0.27–2.03 MJ/kg	Only dust	6–7
Chemical (solvolysis, electrochemical and other depolymerization methods)	90–450 °C/Atmospheric pressure −35 MPa	19.2–90 MJ/kg	Solvents such as alcohols, acids and bases, and heat	3–4
Thermal (pyrolysis and fluidized bed)	400–700 °C/Atmospheric pressure	3–30 MJ/kg	CO_2_, flotsam, and heat	4–8

## Data Availability

Not applicable.
